# Dopamine Reward Prediction Error Responses Reflect Marginal Utility

**DOI:** 10.1016/j.cub.2014.08.064

**Published:** 2014-11-03

**Authors:** William R. Stauffer, Armin Lak, Wolfram Schultz

**Affiliations:** 1 Department of Physiology, Development, and Neuroscience, University of Cambridge, Downing Street, Cambridge CB2 3DY, UK

## Abstract

**Background:**

Optimal choices require an accurate neuronal representation of economic value. In economics, utility functions are mathematical representations of subjective value that can be constructed from choices under risk. Utility usually exhibits a nonlinear relationship to physical reward value that corresponds to risk attitudes and reflects the increasing or decreasing marginal utility obtained with each additional unit of reward. Accordingly, neuronal reward responses coding utility should robustly reflect this nonlinearity.

**Results:**

In two monkeys, we measured utility as a function of physical reward value from meaningful choices under risk (that adhered to first- and second-order stochastic dominance). The resulting nonlinear utility functions predicted the certainty equivalents for new gambles, indicating that the functions’ shapes were meaningful. The monkeys were risk seeking (convex utility function) for low reward and risk avoiding (concave utility function) with higher amounts. Critically, the dopamine prediction error responses at the time of reward itself reflected the nonlinear utility functions measured at the time of choices. In particular, the reward response magnitude depended on the first derivative of the utility function and thus reflected the marginal utility. Furthermore, dopamine responses recorded outside of the task reflected the marginal utility of unpredicted reward. Accordingly, these responses were sufficient to train reinforcement learning models to predict the behaviorally defined expected utility of gambles.

**Conclusions:**

These data suggest a neuronal manifestation of marginal utility in dopamine neurons and indicate a common neuronal basis for fundamental explanatory constructs in animal learning theory (prediction error) and economic decision theory (marginal utility).

## Highlights

• Monkeys’ risk attitudes depend on reward magnitude
• Numerical utility functions are derived from choices under risk
• Dopamine responses depend on the slope of the utility function
• Dopamine reward responses can train risk preferences

## Teaser

Stauffer et al. show that dopamine prediction error responses code marginal utility, a fundamental economic variable that reflects risk preferences. They measure utility functions and relate dopamine reward responses to the utility functions’ first derivatives. The dopamine responses track both increasing and decreasing marginal utility.

## Introduction

The St. Petersburg paradox famously demonstrated that economic choices could not be predicted from physical value. Bernoulli’s enduring solution to this paradox illustrated that decision makers maximized the satisfaction gained from reward, rather than physical value (wealth) [1]. In modern economic theory, the concept of satisfaction was demystified and formalized as “utility.” Utility functions are mathematical representations of subjective value, based on observable choice behavior (rather than unobservable satisfactions) [2]. In expected utility theory, the quantitative relationship between utility and physical value, *U(x)*, can be reconstructed from choices under risk [3]. Such “von-Neumann and Morgenstern” (vNM) utility functions are cardinal, in the strict sense that they are defined up to a positive affine (shape-preserving) transformation [4], in contrast to ordinal utility relationships that are only defined up to a monotonic (rank-preserving) transformation [2]. Thus, the shapes of vNM utility functions are unique, and this formalism permits meaningful approximation of marginal utility—the additional utility gained by consuming additional units of reward—as the first derivative, *dU/dx* [5]. Despite considerable progress demonstrating that numerous brain structures are involved in economic decision-making [6, 7, 8, 9, 10, 11, 12, 13, 14, 15, 16, 17], no animal neurophysiology study has investigated how neurons encode the nonlinear relationship between utility and physical value, as defined by expected utility theory. Most importantly, measurement of neuronal reward responses when utility functions are defined with regard to physical value could provide biological insight regarding the relationship between the satisfaction experienced from reward and the utility function defined from choices.

Midbrain dopamine neurons code reward prediction error, a value interval important for learning [18, 19, 20]. Learning models that faithfully reproduce the actions of dopamine neurons tacitly assume the coding of objective value [18, 21]. However, the dopamine signal shows hyperbolic temporal discounting [10] and incorporates risk and different reward types onto a common currency scale [17]. Prediction error and marginal utility both represent a value interval, and both assume a reference state (prediction and current wealth or rational expectation [22, 23], respectively) and a gain or loss relative to that state. Therefore, the dopamine prediction error signal could be an ideal substrate for coding marginal utility.

Here, we sought to define utility as a function of physical value using risky choices and investigate whether dopamine reward responses reflected the marginal utility calculated from the utility function. We used a classical method for measuring vNM utility functions (the “fractile” procedure) that iteratively aligns gamble outcomes with previously determined points on the utility axis [24, 25]. This procedure resulted in closely spaced estimates of the physical reward amounts mapped onto predefined utilities. The data were fit with a continuous utility function, *U(x)* [24], and the marginal utility was computed as the first derivative, *dU/dx*, of the fitted function [5]. We then recorded dopamine responses to gambles and outcomes and related them to the measured utility function. The absence of common anchor points makes intersubjective utility comparisons generally implausible; therefore, we did not average behavioral and neuronal data across the individual animals studied.

## Results

### Experimental Design and Behavior

**Figure 1:**
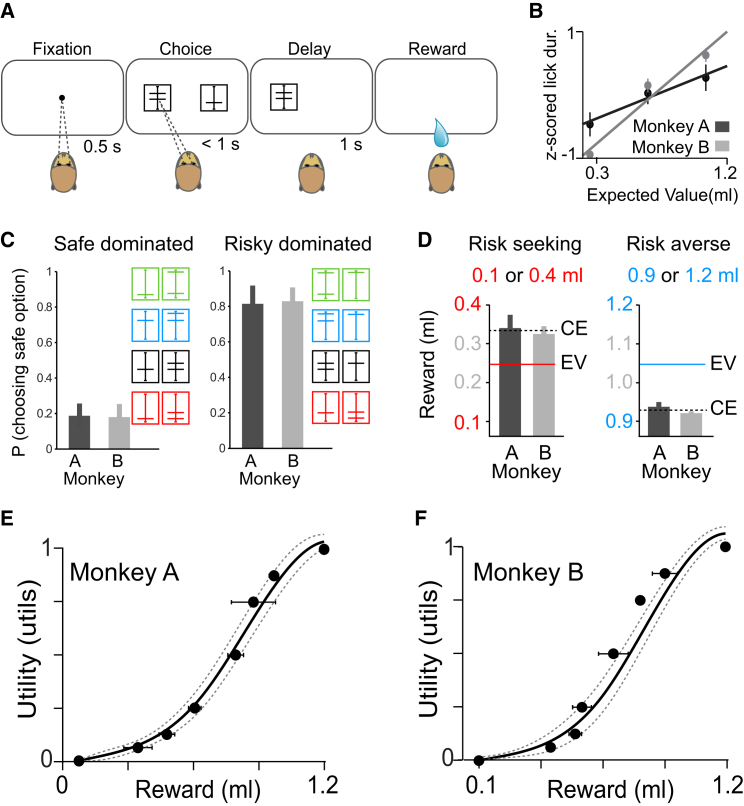
Behavioral Task and Utility Function (A) Choice between safe reward and gamble. The cues indicated safe or risky options with one or two horizontal bars, respectively. (B) Normalized lick duration was correlated positively with the expected value (EV) of the gamble. Error bars indicate the SEM across behavioral sessions. (C) Probabilities of behavioral choices for dominated (left) and dominating (right) safe options. Error bars indicate the SEM from averages across the four choice sets shown to the right. (D) Average CEs of gambles with different EVs in monkeys A and B. Left: risk seeking (CE > EV) with low-value gambles (0.1 or 0.4 ml, red; p < 0.001 and p < 10^−6^, in monkeys A and B, respectively; t test). Right: risk avoidance (CE < EV) with high-value gambles (0.9 or 1.2 ml, blue; p < 10^−7^, both animals). Error bars indicate the SEM across PEST sessions. (E and F) Behavioral utility function measured in monkeys A and B (shown in E and F, respectively) from CEs of binary, equiprobable gambles, using the fractile method one to three times per day. The curve indicates the average best-fit function obtained from cubic splines across measurement days, and the dashed lines insicate ±1 SD across days (Experimental Procedures). The data points represent the mean CEs for one example day of testing (±1 SD). See also Figure S1.

Two monkeys made binary choices between gambles and safe (riskless) reward (Figure 1A). The risky cue predicted a gamble with two equiprobable, no-zero amounts of juice (each p = 0.5), whereas the safe cue was associated with a specific amount of the same juice. The cues were bars whose vertical positions indicated juice amount (see the Experimental Procedures). Both animals received extensive training with >10,000 trials in each gamble. The animals’ lick durations correlated positively with the value of the gambles (Figure 1B). To ascertain whether the animals fully understood the predicted gambles’ values and meaningfully maximized utility during choices under risk, we tested first-order stochastic dominance in choices between a safe reward and a gamble whose low or high outcome equaled the safe reward (Figure S1A available online) [2]. With all four gambles tested, both animals avoided the low safe reward and preferred the high safe reward to the gamble (Figure 1C). Indeed, the values of both the gamble and the safe option significantly affected the animals’ choices on every trial (p < 0.001 for both variables in both animals; logistic regression), and neither animal exhibited a significant side bias (p > 0.5, both animals; logistic regression). Thus, the animals appropriately valued the cues, and their choices followed first-order stochastic dominance.

Risk exerts an important influence on utility; it enhances utility in risk seekers and reduces utility in risk avoiders. To assess the animals’ risk attitudes, we measured the amount of safe reward that led to choice indifference (“certainty equivalent,” CE), for a low and high expected value (EV) gamble. We employed an adaptive psychometric procedure that used the animal’s choice history to present safe options around the indifference point (parameter estimation by sequential testing, PEST; Figure S1B) [26]. The monkeys were risk seeking for a gamble between small rewards (0.1 ml, p = 0.5 and 0.4 ml, p = 0.5), indicated by CEs significantly larger than the EV (Figure 1D, left). However, monkeys were risk averse for a gamble between larger rewards (0.9 ml, p = 0.5 and 1.2 ml, p = 0.5), where the measured CEs were significantly smaller than the gamble EV (Figure 1D, right). To ensure that the animals were maximizing value on every trial, rather than exploiting the adaptive nature of the psychometric algorithm, we verified these results using an incentive-compatible choice procedure in which the current choice options were selected independently of the animals’ previous choices (see the Experimental Procedures). Again, both animals were risk seeking for the small EV gamble but risk averse for the large one (Figure S1C). Thus, the monkeys exhibited risk seeking and risk avoidance depending on reward size, demonstrating a magnitude effect similar to that of human decision makers [27, 28].

To obtain utility functions across a broad range of experimentally reasonable, nonzero juice amounts (0.1–1.2 ml), we used the “fractile” method that iteratively aligns the gamble outcomes to previously determined CEs (Figures S1D and S1E and Experimental Procedures) [24, 25]. For instance, on one particular day, the measured CE for the gamble (0.1 ml, p = 0.5 and 1.2 ml, p = 0.5) was 0.76 ml (Figure S1D, step 1). We used this CE as an outcome to construct two new gambles (0.1 ml, p = 0.5 and 0.76 ml, p = 0.5; 0.76 ml, p = 0.5 and 1.2 ml, p = 0.5). We then measured the CEs for these two new gambles (Figure S1D, steps 2 and 3) and used those measurements to further bisect intervals on the utility axis. This iterative procedure resulted in progressively finer grained gambles, leading to closely spaced CEs for the entire range of 0.1–1.2 ml that mapped onto a numeric utility axis with an arbitrarily chosen origin and range of 0 and 1 util, respectively (Figure S1E). The close spacing of the CEs permitted an estimation of a continuous utility function [24]. To estimate an underlying function without prior assumptions regarding its form, we fit piecewise polynomial functions to the measured CEs (cubic splines with three knots). In both animals, the functions were convex at low juice amounts (indicating risk seeking) and became linear (risk neutral) and then concave (risk avoiding) as juice amounts increased (Figures 1E and 1F). Where the utility function was convex (Figures 1E and 1F), the animals consistently selected more risky options (Figure 1D, left), and where the utility function was concave (Figures 1E and 1F), the animals consistently selected the less risky options (Figure 1D, right). Thus, the risk attitudes inferred from the curvatures of the utility functions confirmed and substantiated the risk attitudes nonparametrically derived from comparison of CEs with EVs. Moreover, the animals’ choices depended neither on the previous outcome (p > 0.5, both animals; logistic regression), nor on accumulated reward over a testing day (p = 0.7 and p = 0.09 for monkeys A and B, respectively; logistic regression). Taken together, these results demonstrated a specific nonlinear subjective weighting of physical reward size that was relatively stable throughout testing.

**Figure 2:**
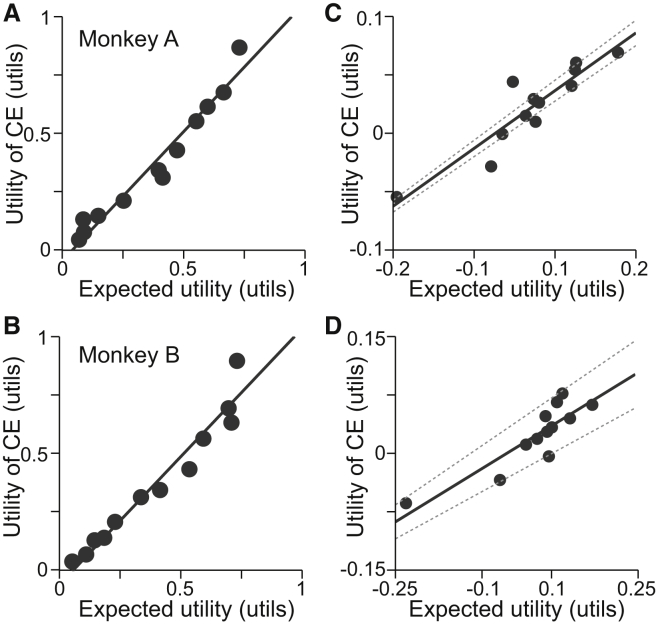
Utility Functions Predicted Risky Choice Behavior (A and B) Out-of-sample predictions for 12 new gambles (Table S1) not used for constructing the utility functions for monkeys A and B (shown in A and B, respectively). All expected utilities were derived from functions in Figures 1E and 1F (monkeys A and B, respectively). The black line represents the fit of a Deming regression. (C and D) Same data as in (A) and (B), but with the EVs removed from the predicted and measured values. The solid line represents the fit of a Deming regression, and dashed lines indicate the 95% confidence interval from the regression. See also Table S1.

To empirically test the particular shape of the constructed utility functions, we investigated how well it predicted the CEs of gambles not used for its construction [24, 29]. We used the measured utility function to calculate the expected utilities (EUs) for 12 new binary, equiprobable gambles with outcomes between 0.1 and 1.2 ml (Table S1) and behaviorally measured the CEs of the new gambles. The calculated EUs predicted well the utilities of the measured CEs (Figures 2A and 2B for monkeys A and B, respectively; Deming regression), suggesting that the utility functions were valid within the range of tested reward, yet this relationship could have been driven by the EV. To better distinguish the predictive power of the nonlinearity in the utility function, we removed the linear EV component from the observed and predicted values. The regressions on the residuals demonstrated a powerful contribution of the curvature of the measured utility functions to explaining choice behavior (Figures 2C and 2D for monkeys A and B, respectively; Deming regression). Thus, the nonlinear shape of the constructed utility function explained choices better than linear physical value. These results provided empirical evidence for the specific shape of the function and suggested that the measured utility functions were unique up to a shape-preserving (i.e., positive affine) transformation. The quasicontinuous nature of the utility function was confirmed in gambles varying reward probability in small steps (Figures S1F and S1G and Supplemental Experimental Procedures). Importantly, this separately measured utility function on the restricted reward range (0.0 to 0.5 ml) did not reflect the same overall shape as the functions measured between 0.1 and 1.2 ml (first convex, then linear, then concave). Rather, the restricted utility function only reflected the convex initial segment of the utility function measured from 0.1 to 1.2 ml. This result suggested that the shape of the overall utility functions (Figures 1E and 1F) did not result from value normalization around the mean. Taken together, these results document that numerically meaningful, quasicontinuous utility functions can be derived in monkeys. Therefore, we used the first derivative of this continuous function to estimate marginal utility.

### Dopamine Responses to Gamble Outcomes

**Figure 3:**
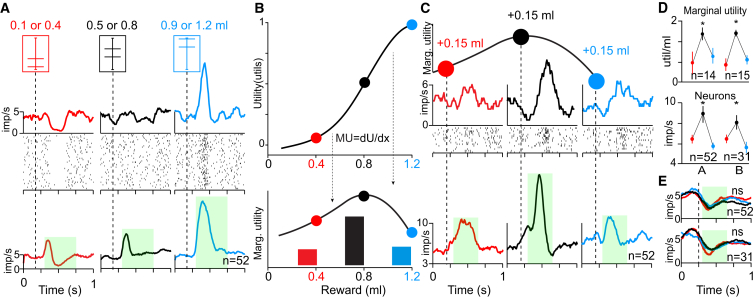
Dopamine Prediction Error Responses Reflect Marginal Utility of Reward (A) Top: three cues, each predicting a binary gamble involving two juice amounts. Middle: single-neuron example of dopamine response to cues shown above (p < 0.001, rho = 0.41; Pearson’s correlation). Bottom: population dopamine response of all recorded neurons (n = 52) in monkey A to cues shown above (p < 0.0001, rho = 0.44; Pearson’s correlation; see Figure S3B for population response in monkey B). The fixation spot that started the trial predicted value equal to the mean value of the three pseudorandomly alternating gambles. Thus, the depression with the low-value gamble reflects the negative prediction error (left), and the activation with the high-value gamble reflects the positive prediction error (right). Despite the short latency activation in the population response to the small gamble, the average response over the whole analysis window (indicated in green) was significantly depressed. (B) Marginal utility as first derivative of nonlinear utility function. The continuous marginal utility function (*dU/dx*; solid black line, bottom) is approximated from utility function (top). Red, black, and blue bars (bottom) represent discrete marginal utilities in three 0.15 ml intervals corresponding to positive prediction errors in gambles shown in at the top of (A). (C) Top: single-neuron PSTHs and rastergrams of positive prediction error responses to the larger reward from the three gambles shown in (A) (0.4, 0.8, and 1.2 ml). Positive prediction errors (0.15 ml) are shown in red, black, and blue above the PSTHs. Data are aligned to prediction errors generated by liquid flow extending beyond offset of smaller reward in each gamble (see Figure S3G). Bottom: population PSTH across all recorded neurons (n = 52) from monkey A to the larger reward from the three gambles shown in (A) (0.4, 0.8, and 1.2 ml). Data are aligned to prediction errors generated by liquid flow extending beyond offset of smaller reward in each gamble (see Figure S3G). (D) Correspondence between marginal utility (top) and neuronal prediction error responses (bottom) in the three gambles tested. Top: marginal utilities associated with receiving larger outcome of each gamble were averaged from 14 and 15 sessions, in monkeys A and B, respectively (approximated by the averaged slope of utility function between predicted and received reward). Asterisks indicate significant differences (p < 0.00001, post hoc Tukey-Kramer after p < 0.0001 Kruskal-Wallis test, both animals). Error bars indicate the SDs across testing sessions. Bottom: average population responses in green analysis window shown in (C). Asterisks indicate significant differences (p < 0.01, post hoc t test with Bonferroni correction after p < 0.00002 and p < 0.008, one-way ANOVA in monkeys A and B, respectively; p < 0.05 in 27 of 83 single neurons, t test). Error bars indicate the SEMs across neurons. (E) Population PSTHs of negative prediction error responses to the smaller reward from the three gambles shown in (A) (0.1, 0.5, and 0.9 ml) in monkeys A (top) and B (bottom). ns, not significantly different from one another. Data are aligned to prediction errors generated by liquid flow stopping (dashed lines). Light-green shaded boxes indicate the analysis windows in (A), (C), and (E). See also Figures S2 and S3.

We investigated the coding of marginal utility by dopamine responses to reward prediction errors, defined as *reward − prediction*. Although we (necessarily) used a choice task to measure utility functions, we examined dopamine reward responses in a nonchoice task. The subtrahend in the previous equation (*prediction*) is not uniquely defined in a choice context; the prediction can be based on some combination of offer values [21]. Therefore, we recorded the electrophysiological responses of 83 typical midbrain dopamine neurons (Figure S2, Experimental Procedures, and Supplemental Experimental Procedures) after extensive training in a nonchoice task (Figure S3A; >10,000 trials/gamble). The animal fixated on a central spot and then was shown one of three specific bar cues predicting a binary, equiprobable gamble between specified juice rewards (0.1 ml, p = 0.5 and 0.4 ml, p = 0.5 in red; 0.5 ml, p = 0.5 and 0.8 ml, p = 0.5 in black; 0.9 ml, p = 0.5 and 1.2 ml, p = 0.5 in blue; Figure 3A, top). The corresponding EVs were small, medium, or large (0.25, 0.65, or 1.05 ml, respectively; Figure 3A, top). The stable dopamine responses to the fixation spot reflected the constant overall mean reward value (0.65 ml) predicted by that stimulus (Figures S3C–S3E). The physical prediction error at each cue was defined by the difference between the EV of each gamble and a constant, mean prediction of 0.65 ml set by the fixation spot. Dopamine responses to the predictive cues showed a significant, positive relationship to prediction error in single neurons (Figure 3A, middle; p < 0.001, rho = 0.68; Pearson’s correlation) and the entire recorded populations (Figure 3A, bottom; n = 52 monkey A; p < 0.0001 in both animals; rho = 0.57 and rho = 0.75 in monkeys A and B, respectively; Pearson’s correlation; see Figure S3B for monkey B population data, n = 31), suggesting that the substantial experience of the animals had induced appropriate neuronal processing of the relative cue values.

To identify the nature of the relationship between dopamine neurons and utility, we inspected their responses to the prediction errors generated by the individual outcomes of the gambles delivered 1.5 s after the respective cues. The prediction errors had identical magnitudes in all three gambles (±0.15 ml), but each gamble was aligned to a different position of the previously assessed nonlinear utility functions (Figure 3B, top). The first derivative of the nonlinear utility function (marginal utility) was significantly larger around the medium gamble compared with the two other gambles (Figure 3B, bottom). Strikingly, the dopamine responses to 0.8 ml of juice in the medium gamble dwarfed the prediction error responses after 0.4 or 1.2 ml in their respective gambles (effect sizes compared to baseline neuronal activity = 1.4 versus 0.7 and 0.6, respectively; p < 0.005; Hedge’s g). The neuronal responses thus followed the inverted U of marginal utility and reflected the slope of the utility function in single neurons (Figure 3C, middle; p < 0.05, rho = 0.41; Pearson’s correlation) and the entire populations in monkeys A (Figures 3C and 3D, bottom; n = 52) and B (Figure 3D, bottom; n = 31) (p < 10^−9^, rho = 0.44; Pearson’s correlation, both animals). In the linear part of the utility function, where the slopes were steeper and marginal utilities significantly higher (Figure 3D, top; p < 0.00001 post hoc Tukey-Kramer after p < 0.0001 Kruskal-Wallis test, both animals), the dopamine responses were significantly stronger, but in the convex and concave parts of the utility functions, where the slopes were shallower and marginal utilities smaller, the responses were also smaller (Figure 3D, bottom; p < 0.01, post hoc t test with Bonferroni correction after p < 0.00002 and p < 0.008, one-way ANOVA in monkeys A and B, respectively; p < 0.05 in 27 of 83 single neurons, two-sided t test between responses to large [black] and small [red and blue] marginal utilities). Although negative prediction error responses were significantly correlated with marginal disutility in 14 individual neurons (p < 0.05; Pearson’s correlation), this relationship failed to reach significance in the population of 52 and 31 neurons of monkeys A and B, respectively (Figure 3E; p > 0.4 and p > 0.2; Pearson’s correlation), perhaps because of the naturally low baseline impulse rate and accompanying small dynamic range. When we analyzed positive and negative prediction error responses together, there was a strong correlation with marginal utility (44 single neurons, p < 0.05; population, p < 10^−7^ and p < 10^−15^, rho = 0.3 and rho =0.5, in monkeys A and B, respectively; Pearson’s correlation). However, this combined analysis didn’t account for the (approximately 5-fold) asymmetric dynamic range of dopamine neurons in the positive and negative domains and therefore should be taken with caution.

To confirm that the particular nature of the temporal prediction errors in the experiment did not explain the observed neuronal utility coding, we used temporal difference (TD) models and ruled out other possibilities, such as differential liquid valve opening durations (Supplemental Experimental Procedures and Figures S3F–S3J). Importantly, we are aware of no simple subjective value measure for reward size that could explain the nonlinear dopamine responses to receiving an extra 0.15 ml. Rather, the prediction on the utility scale and the corresponding slope of the utility function was necessary to explain the observed responses. Thus, these data strongly suggest that the dopamine prediction error response coded the marginal utility of reward.

### Dopamine Responses to Unpredicted Reward

**Figure 4:**
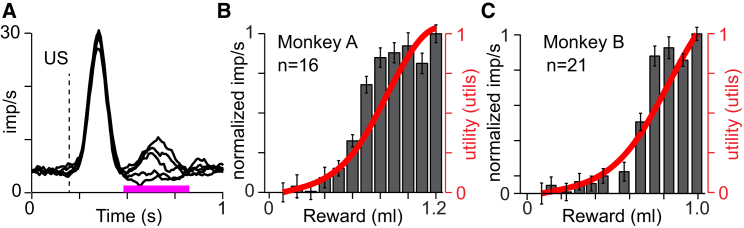
Responses to Unpredicted Reward Reflect Marginal Utility (A) Population histogram of dopamine neurons from monkey A (n = 16). The early component was statistically indistinguishable, but the late component (indicated by the pink horizontal bar) was reward magnitude dependent. For display purposes, the neuronal responses to five reward sizes (rather than 12) are shown. (B and C) Average population dopamine responses to different juice amounts, measured in the time window shown in (A) (pink bar) and normalized to 0 and 1, for monkeys A and B (shown in B and C, respectively). In each graph, the red line (which corresponds to the secondary y axis) shows the utility gained from each specific reward over zero (marginal utility) and is identical to the utility function measured separately in each animal (Figures 1E and 1F). The utility function for monkey B was truncated at the largest reward delivered (to monkey B) in the unpredicted reward task (1.0 ml). n, number of neurons. Error bars indicate the SEMs across neurons. See also Figure S2.

To obtain a more fine-grained neuronal function for comparison with marginal utility, we used 12 distinct reward volumes distributed across the reward range of the measured utility functions (0.1–1.2 ml in monkey A, but monkey B was only tested between 0.1 and 1 ml). Because these rewards were delivered without any temporal structure, explicit cue, or specific behavioral contingencies, the animals could not predict when in time the reward would be delivered, and thus the moment-by-moment reward prediction was constant and very close to zero. Therefore, in contrast to the gambles with their different predicted values (Figure 3), the marginal utility of each unpredicted reward was defined as the interval between the utility of each reward and the constant utility of the moment-by-moment prediction of zero. In this context the marginal utility followed by definition the utility function. We recorded 37 additional neurons (n = 16 and n = 21 in monkeys A and B, respectively) while the animals received one of the 12 possible reward sizes at unpredictable moments in time. The late, differential response component reflected closely the nonlinear increase in marginal utility as reward amounts increased (Figure 4; p < 10^−4^, both animals; rho = 0.94 and rho = 0.97; Pearson’s correlation). Despite some apparent deviations, the difference between the neuronal responses and the utility functions were not significantly different from zero (p > 0.1 and p > 0.2, in monkeys A and B, respectively; t test). Thus, the dopamine responses to unpredicted reward reflected marginal utility.

### Neuronal Teaching Signal for Utility

**Figure 5:**
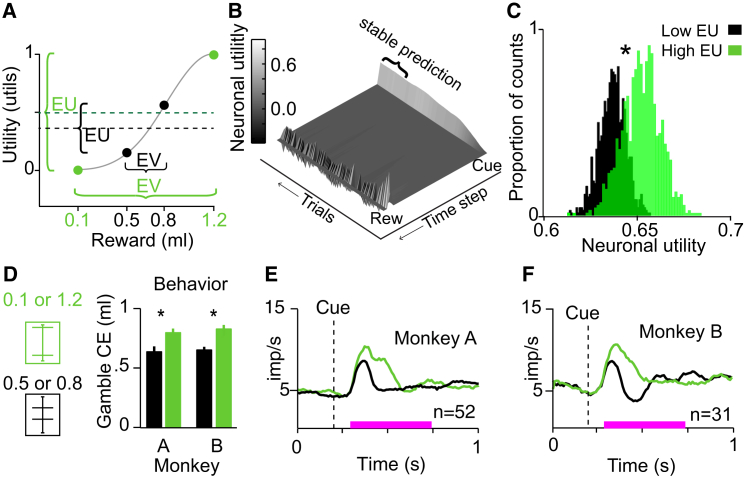
Modeled and Recorded Expected Utility Responses (A) Gambles for reinforcement modeling. Two gambles (0.1 ml, p = 0.5 and 1.2 ml, p = 0.5 [green]; 0.5 ml, p = 0.5 and 0.8 ml, p = 0.5 [black]) had equal expected value (EV = 0.65 ml) but different risks. The gambles were aligned onto the previously established utility function (Figure 1F) and yielded higher (green) and lower (black) expected utility (EU; horizontal dashed lines). (B) A TD model learned to predict gamble utility from dopamine responses. The surface plot represents the prediction error term from a TD model (eligibility trace parameter λ = 0.9) trained on the neuronal responses to 0.5 or 0.8 ml of juice. The normalized population responses (from the window defined by the pink bar; Figure 4A) were delivered as outcomes (Rew), and trained a cue response (Cue). The “cue” response fluctuated due to the pseudorandom outcome delivery. “Stable prediction” indicates the phase after initial learning when the responses fluctuated about a stable mean, and the average of this value was used in (C) (where it was defined as the last 200 of 1,000 trials; here we show 150 trials for display purposes). (C) Histograms of learned TD prediction errors reflect the expected utility of gambles when trained on neuronal responses. Each histogram is comprised of 2,000 data points that represent the predicted gamble value (high expected utility gamble in green versus low expected utility gamble in black, defined in A) after training (shown in B) with the neuronal responses from monkey A (Figure 4A) to the respective gamble outcomes (^∗^p < 10^−254^; t test.) (D) Behaviorally defined certainty equivalents reflect the expected utilities predicted in (A). Higher CE for riskier gamble suggests compliance with second-order stochastic dominance. Error bars indicate the SEMs across CE measurements. (E and F) Stronger dopamine responses to higher expected utility gamble (green) compared to lower expected utility gamble (black) in monkeys A (shown in E; p < 0.004, t test) and B (shown in F; p < 0.02, t test), consistent with second-order stochastic dominance. The pink bar shows the analysis time window. n, number of neurons. See also Figures S2 and S4.

Dopamine prediction error responses are compatible with teaching signals defined by TD reinforcement models [18, 21]. TD models learn a value prediction from outcomes; we therefore tested whether the value prediction a TD model learned from an animal’s dopamine responses would reflect the expected utility defined by the animal’s choice behavior. To do so, we constructed two gambles (0.5 ml, p = 0.5 and 0.8 ml, p = 0.5; 0.1 ml, p = 0.5 and 1.2 ml, p = 0.5) with identical EV but different expected utilities (Figure 5A) and took the dopamine responses to those four outcomes (Figure 4B) as the inputs to our models. We trained two models to predict the value of the two gambles, separately. The first model was trained with the population dopamine responses to either 0.5 or 0.8 ml of reward, delivered in pseudorandom alternation with equal probability of p = 0.5 (Figure 5B). The second model was trained with the dopamine responses to 0.1 or 1.2 ml (both p = 0.5). Each learning simulation was run for 1,000 trials, and each simulation was repeated 2,000 times to account for the pseudorandom outcome schedule. The TD model trained on the dopamine responses to 0.1 or 1.2 ml acquired a learned value prediction that was significantly larger than the learned value prediction of the TD model trained on responses to 0.5 or 0.8 ml (Figure 5C). Thus, the differential TD model responses to the cues correctly reflected the expected utility of the gambles and thus the risk attitudes of the animals. Similarly, TD models trained on dopamine responses from monkey B (Figure 4C) also learned an expected-utility-like prediction when the same procedure was repeated with different gambles (0.35 ml, p = 0.5 and 0.75 ml, p = 0.5; 0.1 ml, p = 0.5 and 1.0 ml, p = 0.5) (Figures S4A and S4B). Although these data cannot show that dopamine neurons provide the original source for utility functions in the brain, these modeling results demonstrate that dopamine responses could serve as an effective teaching signals for establishing utility predictions of risky gambles and training economic preferences.

### Neuronal Cue Responses Reflect Expected Utility

To examine whether risk was incorporated into the neuronal signals in a meaningful fashion consistent with expected utility theory and predicted by the modeling results, we examined dopamine responses to the same two gambles employed for the reinforcement model. The riskier gamble was a mean preserving spread of the less risky gamble, thus removing any effects of returns [30]. As calculated from the extensively tested utility function, the riskier gamble had a higher expected utility (Figure 5A) and second-order stochastically dominated the less risky gamble in risk seekers [31]. Accordingly, both monkeys reported a higher CE for the riskier gamble compared to the less risky one (measured in choices; Figure 5D), and dopamine responses to the cues were significantly stronger for the riskier compared to the less risky gamble (Figures 5E and 5F, green versus black). Thus, both the behavior and the neuronal responses were compatible with second-order stochastic dominance, suggesting meaningful incorporation of risk into utility at both the behavioral and neuronal level. Consistent with the modeled cue responses (Figure 5), the dopamine responses appeared to reflect the expected utilities derived from the measured utility function, rather than the probability or the EV of the gambles. Importantly, the dopamine utility responses to the cue did not code risk independently of value; the responses were similar between a gamble with considerable risk and a safe reward when the two had similar utility (Figure S4C). Thus, the observed behavioral and neuronal responses demonstrated that the dopamine neurons meaningfully incorporated risk into utility and suggested that dopamine neurons support the economic behavior of the animals.

### Dopamine Responses Comply with First-Order Stochastic Dominance

**Figure 6:**
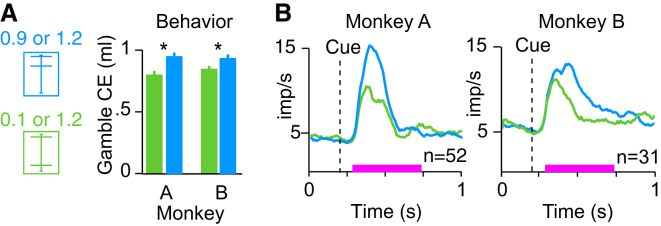
First-Order Stochastic Dominance Dopamine responses comply with first-order stochastic dominance. (A) Two binary, equiprobable gambles (left) had identical upper outcomes; they varied in lower outcomes which defined the value difference between the gambles and thus the first-order stochastic dominance. Measured CEs were larger for the gamble with larger EV (p < 0.02, right, both animals, t test). Error bars indicate the SEMs across CE measurements. (B) Neuronal population responses were larger to the stochastically dominant gamble (blue versus green) (p < 0.01, both animals, t test). These response differences suggest coding of expected utility rather than upper bar height or value of better outcome (which were both constant between the two gambles). Pink horizontal bars show neuronal analysis time window. n, number of neurons. See also Figure S2.

As the animals’ choices complied with first-order stochastic dominance (Figures 1C and S1A), we examined whether dopamine cue responses were consistent with this behavior. We examined responses to binary, equiprobable gambles with identical upper outcomes but different lower outcomes (Figure 6A). With any strictly positive monotonic value function, including our established utility functions (Figures 1E and 1F), the lower outcomes, in the face of identical upper outcomes, determine the preference ordering between the two gambles [2]. Both monkeys valued the cues appropriately (Figure 6A, right). Accordingly, dopamine responses to cues were significantly larger for the more valuable gamble and didn’t simply code upper bar height (Figure 6B). Thus, as with the animals’ choices, the dopamine neurons followed first-order stochastic dominance, suggesting appropriate neuronal processing of economic values.

## Discussion

These data demonstrate that dopamine prediction error responses represent a neuronal correlate for the fundamental behavioral variable of marginal utility. The crucial manipulation used here was the measurement of quantitative utility functions from choices under risk, using well-established procedures. The measured functions provided a nonlinear numerical function between physical reward amounts and utility whose shape was meaningful. This function permitted meaningful computation of marginal utility as the first derivative. The dopamine prediction error responses to gamble outcomes and to unpredicted reward reflected the marginal utility of reward. The modeling data suggested that the dopamine marginal utility signal could train appropriate neuronal correlates of utility for economic decisions under risk. As prediction error and marginal utility arise from different worlds of behavioral analysis, a dopamine prediction error signal coding marginal utility could provide a biological link between animal learning theory and economic decision theory.

Although previous studies have shown that dopamine cue and reward responses encode a subjective value prediction error [10, 17], perhaps the most interesting aspect of this study is that responses to the reward itself (rather than the cue responses) reflected specifically the first derivative of the measured utility function. There is no a priori reason that they should do so. Economic utility is measured by observed choices, and economic theory is generally agnostic about what happens afterward. For example, risk aversion is commonly attributed to diminishing marginal utility, yet because economists observe choice rather than marginal utility, the explanation of diminishing marginal utility is an “as if” concept. In this study, the dopamine reward responses were directly related to the shape of the utility function defined from risky choices. The difference between the dopamine response magnitudes from one reward to the next larger reward decreased in the risk-avoiding range and increased in the risk-seeking range (Figures 3C, 3D, 4B, and 4C). Thus, the nonlinear neuronal response function to reward provides direct evidence for a biological correlate for marginal utility.

The behavioral compliance with first- and second-order stochastic dominance provided strong evidence that both animals made meaningful economic choices. First-order stochastic dominance dictates what option should be chosen based on the cumulative distribution functions (CDFs) of the offer values [2, 32]. One choice option first-order stochastically dominates a second option when there is nothing to lose by selecting the former (the CDF of the dominating option is always lower to the right; Figure S1A). Therefore, individuals who meet the most basic criteria of valuing larger rewards over smaller rewards should chose the dominating option, just like our monkeys did (Figure 1C). Second-order stochastic dominance dictates the utility ranking of options with equal expected returns but different risk [30]. For risk avoiders (concave utility function), a less risky gamble second-order stochastically dominates a more risky gamble with the same expected return. For risk seekers (convex utility function), the opposite is true [31]. Because second-order stochastic dominance uses utility functions to dictate what gamble should be chosen, adherence to second-order stochastic dominance could be used as a measure of choice consistency. Both of our monkeys were overall risk seekers (indicated by the inflection point of the utility function offset to the right; Figures 1E and 1F), and they both reported a higher CE for gambles with larger risk compared to a gamble with smaller risk but the same expected return (Figure 5D). Together, these two measures indicated that both animals combined objective value with risk in a meaningful way and maximized expected utility.

The ability of the recovered utility functions to predict risky choice behavior provided additional strong evidence that the animals were approximating the behavior of expected utility maximizers. Although traditional expected utility maximizers follow a set of axioms establishing basic rationality [3], it is not always practical or feasible to measure how close actual behavior matches the axioms [29]. Therefore, an accepted test for the shape of measured utility functions is to investigate how well they can predict risky choices [24, 29]. The utility function measured in both animals predicted well the values they assigned to gambles not used for constructing the function (Figures 2C and 2D). Moreover, the utility functions adhered to basic assumptions about how utility functions should behave; specifically, they were positive monotonic, nonsaturating within our reward range and quasicontinuous due to the fine-grained fractile CE assessment. Taken together, these observations suggest that the monkeys made meaningful, value-based choices and that the measured utility functions reflected the underlying preferences.

The convex-concave curvature of the measured utility function differed from the usually assumed concave form but is nevertheless well known in both economic utility theory [27, 28, 33] and animal learning theory [24]. Risk-seeking behavior for small reward has often been observed in monkeys [15, 17, 34, 35, 36], and the increasing risk aversion with increasing reward size nicely mirrored risk sensitivities in humans [37]. By following a proven procedure for recovering vNM utility [24, 25], and because the resulting functions were able to predict preferences for risky gambles [29], our measured utility functions retained important features of cardinal utility. Although the origin and scale of our utility functions were arbitrary, the shape of utility functions measured under risk is unique (defined up to a positive affine transformation) [3, 4]. Utility function with these properties thus allowed estimation of marginal utility and statistically meaningful correlations with naturally quantitative neuronal responses.

Although expected utility theory is the dominant normative theory of economic decision-making, it fails to provide an adequate description of actual choice behavior [38, 39, 40]. The Allais paradox in particular demonstrates that human decision makers do not treat probability in a linear fashion [38]. They normally overvalue small probabilities and undervalue large ones [40, 41]. In this study, we selected one probability, p = 0.5, where distortion is minimal [41]. Moreover, because the probability was constant, any probability distortion was also constant and could not influence the curvature of our measured function.

The coding of marginal utility by prediction error responses suggests a common neuronal implementation of these two different terms, which would suit the dual function of phasic dopamine signals. The dopamine prediction error response is a well-known teaching signal [18, 42], yet recent research suggested that dopamine may also influence choice on a trial-by-trial basis [43]. Coding the marginal utility associated with prediction errors would suit both functions well. Decision makers maximize utility rather than objective value; therefore, neurons participating in decision-making should be coding utility rather than objective value. Indeed, many reward neurons in dopamine projection areas track subjective value [7, 11, 15, 16, 44]. The modeling results demonstrated that these dopamine responses would be suitable to update economic value coding in these neurons. Although some values may be computed online rather than learned though trial and error [45], having a teaching signal encoding marginal utility removes the time-consuming transformation from objective value and thus is evolutionary adaptive.

Despite the unambiguously significant marginal utility responses in the positive domain, the negative prediction error responses failed to significantly code marginal disutility. This negative result could stem from the simple fact that the dynamic range in the positive domain is approximately 5-fold greater than the dynamic range in the negative domain. Alternatively, it is possible that the measured utility functions did not correctly capture marginal disutility. Modern decision theories posit that the natural reference point with which to measure gains and losses is predicted wealth [22, 23]. Under this theoretical framework, the received rewards that were smaller than predicted would be considered losses. Utility functions spanning the gain and loss domain are “kinked” at the reference point [40], therefore the marginal disutility would not be the mirror image of the marginal utility. Future studies will investigate this intriguing possibility.

Distinct from the observed adaptation to reward range [46], the current dopamine responses failed to adapt to the different gambles, possibly because of the larger tested reward range and more demanding reward variation. Also distinct from previously observed objective risk coding in dopamine neurons [47], orbitofrontal cortex [35, 48], and anterior dorsal septum [49], the current data, consistent with our previous observations [17], demonstrate the incorporation of risk into value coding in a manner consistent with traditional theories of utility. Risk affects value processing in human prefrontal cortex in a manner compatible with risk attitude [50]. The current data provide a neuronal substrate for this effect by linking it to a specific neuronal type and mechanism.

## Experimental Procedures

### Animals and General Behavior

Two male rhesus monkeys (*Macaca mulatta*; 13.4 and 13.1 kg) were used for all studies. All experimental protocols and procedures were approved by the Home Office of the United Kingdom. A titanium head holder (Gray Matter Research) and stainless steel recording chamber (Crist Instruments and custom made) were aseptically implanted under general anesthesia before the experiment. The recording chamber for vertical electrode entry was centered 8 mm anterior to the interaural line. During experiments, animals sat in a primate chair (Crist Instruments) positioned 30 cm from a computer monitor. During behavioral training, testing and neuronal recording, eye position was monitored noninvasively using infrared eye tracking (ETL200; ISCAN). Licking was monitored with an infrared optosensor positioned in front of the juice spout (V6AP; STM Sensors). Eye, lick, and digital task event signals were sampled at 2 kHz and stored at 200 Hz (eye) or 1 kHz. Custom-made software (MATLAB, Mathworks) running on a Microsoft Windows XP computer controlled the behavioral tasks.

### Behavioral Task and Analysis

The animals associated visual cues with reward of different amounts and risk levels. We employed a task involving choice between safe (riskless) and risky reward for behavioral measurements and a nonchoice task for the neuronal recordings. The cues contained horizontal bars whose vertical positions indicated the reward amount (between 0.1 and 1.2 ml in both animals). A cue with a single bar indicated a safe reward, and a cue with double bars signaled an equiprobable gamble between two outcomes indicated by their respective bar positions.

Each trial began with a fixation spot at the center of the monitor. The animal directed its gaze to it and held it there for 0.5 s. Then the fixation spot disappeared. In the choice task (Figure 1A), one specific gamble cue and a safe cue appeared to the left and right of the fixation spot, pseudorandomly varying between the two positions. The animal had 1 s to indicate its choice by shifting its gaze to the center of the chosen cue and holding it there for another 0.5 s. Then the unchosen cue disappeared while the chosen cue remained on the screen for an additional 1 s. The chosen reward was delivered at offset of the chosen cue by means of a computer controlled solenoid liquid valve (0.004 ml/ms opening time).

We performed a binomial logistic regression to assess the effect on choice (for gamble or safe option) of the following factors: gamble value, safe value, accumulated daily reward, prior outcome (better or worse than predicted for the gamble and as predicted for the safe option), and position on the screen.

### Estimation of CEs using PEST

To measure CEs and to construct utility curves, we used PEST. We assessed the amount of blackcurrant juice that was subjectively equivalent to the value associated with each gamble (Figure 1D). The rules governing the PEST procedure were adapted from Luce [26]. Each PEST sequence consisted of several consecutive trials during which one constant gamble was presented as a choice option against the safe reward. On the initial trial of a PEST sequence, the amount of safe reward was chosen randomly from the interval 0.1 to 1.2 ml. Based on the animal’s choice between the safe reward and gamble, the safe amount was adjusted on the subsequent trial. If the animal chose the gamble on trial *t*, then the safe amount was increased by ε on trial *t* + 1. However, if the animal chose the safe reward on trial *t*, the safe amount was reduced by ε on trial *t* + 1. Initially, ε was large. After the third trial of a PEST sequence, ε was adjusted according to the doubling rule and the halving rule. Specifically, every time two consecutive choices were the same, the size of ε was doubled, and every time the animal switched from one option to the other, the size of ε was halved. Thus, the procedure converged by locating subsequent safe offers on either side of the true indifference value and reducing ε until the interval containing the indifference value was small. The size of this interval is a parameter set by the experimenter, called the exit rule. For our study, the exit rule was 20 μl. When ε fell below the exit rule, the PEST procedure terminated, and the indifference value was calculated by taking the mean of the final two safe rewards. A typical PEST session lasted 15–20 trials.

### Incentive Compatible Psychometric Measurement of CEs

To confirm the CEs measured using PEST method, we used a choice task wherein the choice options did not depend on animal’s previous choice (i.e., incentive compatible). We assessed CEs psychometrically from choices between a safe reward and a binary, equiprobable gamble (p = 0.5, each option), using simultaneously presented bar cues for safe reward and gamble. We varied pseudorandomly the safe reward across the whole range of values (flat probability distribution), thus setting the tested values irrespectively of the animal’s previous choices. We thus estimated the probability with which monkeys were choosing the safe reward over the gamble for a wide range of reward magnitudes. We fitted the logistic function of the following form on these choice data:

$$P\left(SafeChoice\right)=1/\left(1+e^{-\left(\alpha +\beta \left(SafeReward\left(ml\right)\right)\right)}\right),$$

where α is a measure of choice bias and β reflects sensitivity (slope). The CE of each gamble was then estimated from the psychometric curve by determining the point on the x axis which corresponded to 50% choice (indifference) in the y axis. As Figure S1C illustrates, for the gamble with low EV (red), the estimated CE was larger than the gamble’s EV, indicating risk seeking. By contrast, for the gamble with high EV (blue), the estimated CE was smaller than the gamble’s EV, indicating risk aversion.

### Constructing Utility Functions with the Fractile Method

We determined each monkey’s utility function in the range between 0.1 and 1.2 ml using the fractile method on binary, equiprobable gambles (each p = 0.5; one example fractile procedure is shown in Figure S1C) [24, 25]. To do so, we first measured the CE of an equiprobable gamble (p = 0.5, each outcome) between 0.1 and 1.2 ml using PEST. The measured CE in the example of Figure S1D was 0.76 ml. Setting of u(0.1 ml) = 0 util and u(1.2 ml) = 1 util results in u(0.76 ml) = 0.5 util. We the used this CE as an outcome to construct two new gambles (0.1 ml, p = 0.5 and 0.76 ml, p = 0.5; 0.76 ml, p = 0.5 and 1.2 ml, p = 0.5) then measured their CEs (Figure S1D, steps 2 and 3), which corresponded to u = 0.25 and 0. 75 util, respectively (Figure S1D, steps 2 and 3). We iterated this procedure, using previously established CE as the outcomes in new gambles, until we had between seven and nine CEs corresponding to utilities of 0, 0.063, 0.125, 0.25, 0.5, 0.75, 0.875, 0.938, and 1.0 util (in the example session shown in Figure S1E, seven points were measured, and the two corresponding to 0.063 and 0.938 were omitted). We fit cubic splines to these data (see below for details) and provided an estimation of the shape of the monkey’s utility function in the range of 0.1 and 1.2 ml for that session of testing.

We repeatedly estimated the utility function of the monkeys over different days of testing (14 and 15 times for monkeys A and B, respectively). For each fractile procedure, we measured each gamble multiple times and used the average CE as the outcome for the next gamble. We then fit the data acquired in each fractile procedure using local data interpolation (i.e., splines, MATLAB SLM tool). We used such fitting in order to avoid any assumption about the form of the fitted function. This procedure fits cubic functions on consecutive segments of the data and uses the least square method to minimize the difference between empirical data and the fitted curve. The number of polynomial pieces was controlled by the number of knots which the algorithm was allowed to freely place on the x axis. We used three knots for our fittings. We restricted the fitting to have a positive slope over the whole range of the outcomes, and we required the function to be weakly increasing based on the fundamental economic assumption that more of a good does not decrease total utility, i.e., the function was nonsatiating in the range used. The fits were averaged together to give the final function (Figures 1E and 1F).

### Neuronal Data Acquisition and Analysis

Dopamine neurons were functionally localized with respect to (1) the trigeminal somatosensory thalamus explored in awake animals and under general anesthesia (very small perioral and intraoral receptive fields, high proportion of tonic responses, 2–3 mm dorsoventral extent), (2) tonically position coding ocular motor neurons, and (3) phasically direction coding ocular premotor neurons in awake animals (Figure S2). Individual dopamine neurons were identified using established criteria of long waveform (>2.5 ms) and low baseline firing (fewer than eight impulses per second). We recorded extracellular activity from 120 dopamine neurons (68 and 52 in monkeys A and B, respectively) during the reward prediction tasks and with unpredicted reward (83 and 37 neurons, respectively). Most neurons that met these criteria showed the typical phasic activation after unexpected reward, which was used as fourth criterion for inclusion. Further details on identification of dopamine neurons are found in the Supplemental Experimental Procedures.

During recording, each trial began with a fixation point at the center of the monitor. The animal directed its gaze to it and held it for 0.5 s. Then the fixation point disappeared and a cue predicting a gamble occurred. Gambles alternated pseudorandomly (see below). The cue remained on the screen for 1.5 s. One of the two possible gamble outcomes was delivered at cue offset. Unsuccessful central fixation resulted in a 6 s timeout. There was no behavioral requirement after the central fixation time had ended. Trials were interleaved with intertrial intervals of pseudorandom durations conforming to a truncated Poisson distribution (λ = 5, truncated between 2 and 8 s.). We normally recorded 150–180 trials per neuron and two to three neurons per day.

Cue presentation order was determined by drawing without replacement from the entire pool of trials that we hoped to record. This procedure ensured that we acquired sufficient trials per condition and made it very difficult to predict which cue would come next. Indeed, we saw no indication in the behavior or neural data (Figure S3) that the animals could predict upcoming cue. The order of unpredicted reward was determined in the same way. In monkey A, we tested reward magnitudes of 0.1, 0.2, 0.3, 0.4, 0.5, 0.6, 0.7, 0.8, 0.9, 1, 1.1, and 1.2 ml. In monkey B, this particular test was done before we knew the final reward range we would establish utility functions for, and so we tested reward magnitudes 0.11, 0.18, 0.22, 0.3, 0.35, 0.44, 0.59, 0.68, 0.75, 0.84, 0.9, and 1 ml.

We analyzed neuronal data in three task epochs after onsets of fixation spot, cue, and juice. We constructed peristimulus time histograms (PSTHs) by aligning the neuronal impulses to task events and then averaging across multiple trials. The impulse rates were calculated in nonoverlapping time bins of 10 ms. PSTHs were smoothed using a moving average of 70 ms for display purposes. Quantitative analysis of neuronal data employed defined time windows that included the major positive and negative response components following fixation spot onset (100–400 ms), cue onset (100–550 ms in monkeys A and B), and juice delivery (50–350 ms and 50–450 ms in monkeys A and B, respectively). For the analysis of neuronal response to juice, we aligned the neuronal response in each trial type to the prediction error time for that trial type. Because each gamble was predicting two possible amounts of juice (onset of which occurred 1.5 s after cue), the onset of juice delivery should not generate a prediction error. However, the continuation of juice delivery after the time necessary for the delivery of the smaller possible juice reward should generate a positive prediction error. Thus, the prediction error occurred at the time at which the smaller reward with each gamble (would have) ended (see Figures S3G and S3H). For example, the 0.4 ml juice was the larger outcome of a gamble between 0.1 and 0.4 ml of juice, and hence the dopamine responses were aligned to the prediction error occurring after the solenoid duration that would have delivered 0.1 ml of juice (∼25 ms). Consistent with this, dopamine prediction error responses to reward appeared with longer delay in trials involving gambles with larger EVs (Figure S3I). Analysis of responses to varying sizes of unpredicted juice outside of the task employed a later time window that captured the differential neuronal responses to reward with different sizes (200–500 ms and 300–600 ms in monkeys A and B, respectively). We measured the effect size between the neuronal responses to each reward size versus the baseline firing rate of neurons for Figures 3C and 3D (bottom) with Hedge’s g. Hedge’s g around 0.2, 0.5, and 0.8 indicate small, medium, and large effects, respectively. The confidence intervals for the effect size indicating significant deviation from g = 0 were obtained by bootstrapping with 100,000 permutations.

## Author Contributions

W.R.S., A.L., and W.S. designed the research and wrote the paper. W.R.S. and A.L. collected and analyzed the data.

## Supplemental Information

Document S1. Supplemental Experimental Procedures, Figures S1–S4, and Table S1

Document S2. Article plus Supplemental Information
