# Supplementary material for: Dopamine Reward Prediction Error Responses Reflect Marginal Utility
Source: Curr Biol. 2014 Nov 3;24(21):2491–500. doi: 10.1016/j.cub.2014.08.064 (PMC4228052; doi:10.1016/j.cub.2014.08.064)
Supplement: Document S1. Supplemental Experimental Procedures, Figures S1–S4, and Table S1 [file mmc1.pdf]

Current Biology, Volume *24*

Supplemental Information

Dopamine Reward Prediction Error Responses Reflect Marginal Utility

William R. Stauffer, Armin Lak, and Wolfram Schultz

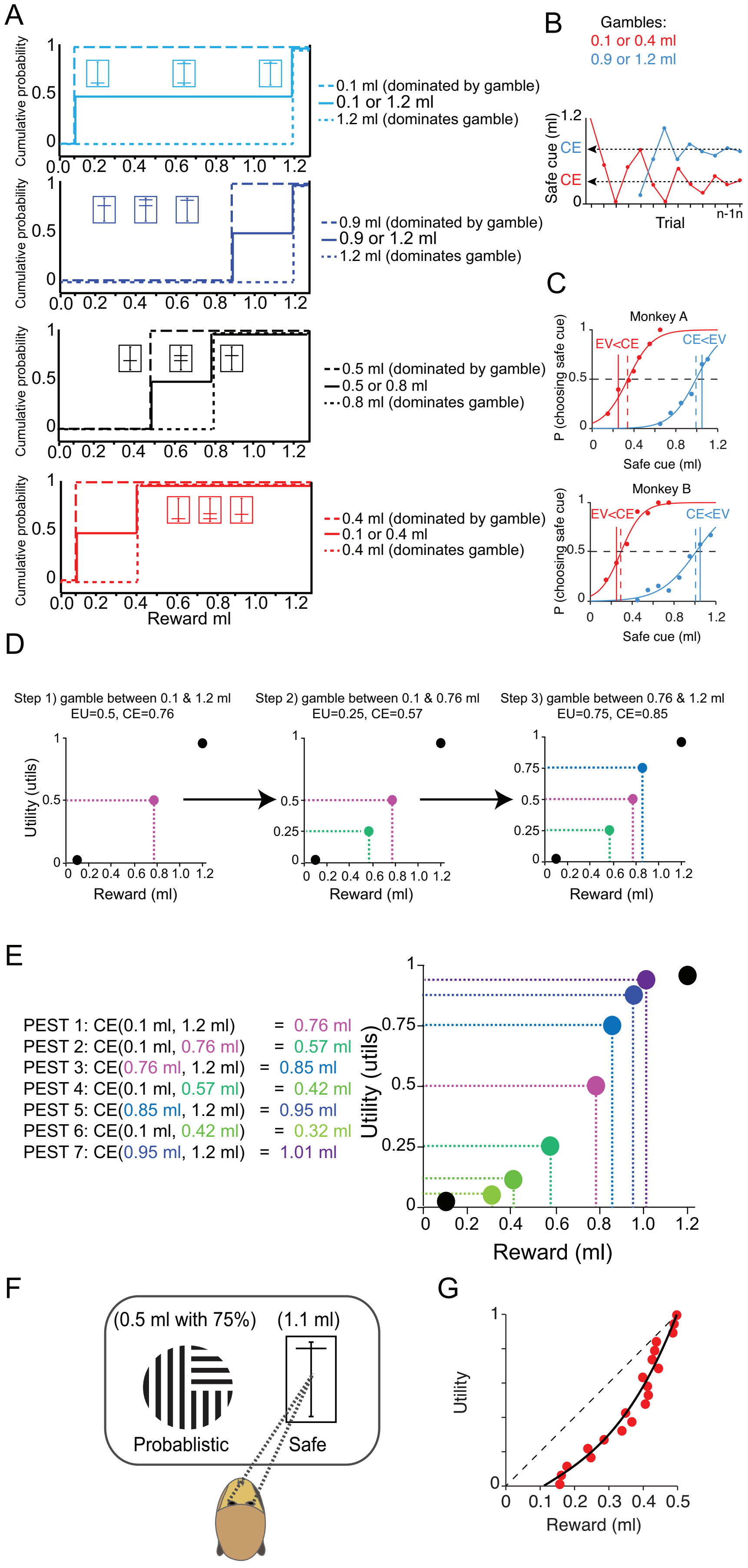

A
Cumulative probability
0.1 ml (dominated by gamble)
0.1 or 1.2 ml
1.2 ml (dominates gamble)
0.9 ml (dominated by gamble)
0.9 or 1.2 ml
1.2 ml (dominates gamble)
0.5 ml (dominated by gamble)
0.5 or 0.8 ml
0.8 ml (dominates gamble)
0.4 ml (dominated by gamble)
0.1 or 0.4 ml
0.4 ml (dominates gamble)
Reward ml
B
Gambles:
0.1 or 0.4 ml
0.9 or 1.2 ml
Safe cue (ml)
CE
CE
Trial
n-1n
C
Monkey A
EV<CE
CE<EV
P (choosing safe cue)
Safe cue (ml)
Monkey B
EV<CE
CE<EV
P (choosing safe cue)
Safe cue (ml)
D
Step 1) gamble between 0.1 & 1.2 ml
EU=0.5, CE=0.76
Step 2) gamble between 0.1 & 0.76 ml
EU=0.25, CE=0.57
Step 3) gamble between 0.76 & 1.2 ml
EU=0.75, CE=0.85
Utility (utils)
Reward (ml)
E
PEST 1: CE(0.1 ml, 1.2 ml) = 0.76 ml
PEST 2: CE(0.1 ml, 0.76 ml) = 0.57 ml
PEST 3: CE(0.76 ml, 1.2 ml) = 0.85 ml
PEST 4: CE(0.1 ml, 0.57 ml) = 0.42 ml
PEST 5: CE(0.85 ml, 1.2 ml) = 0.95 ml
PEST 6: CE(0.1 ml, 0.42 ml) = 0.32 ml
PEST 7: CE(0.95 ml, 1.2 ml) = 1.01 ml
Utility (utils)
Reward (ml)
F
(0.5 ml with 75%)
(1.1 ml)
Probablistic
Safe
G
Utility
Reward (ml)

Figure S1: Behavioral tests (related to Figure 1)

(A) Test of first order stochastic dominance. Four comparisons of cumulative reward distributions for different safe and risky reward options (top to bottom). Interrupted, solid and dotted lines from left to right indicate increasing value and thus stochastic dominance. Insets in each graph show reward magnitude cues (vertical positions of horizontal lines) for safe outcomes and gambles (from left to right: low safe reward with p=1.0, gamble with p=0.5 each outcome, high safe reward with p=1.0). Low safe rewards were set at low gamble outcomes, and high safe rewards were set at high gamble outcomes. Thus each reward of the same magnitude varied in probability (p=1.0 as safe outcome vs. p=0.5 in gamble). For example, for choices between large value safe reward of 1.2 ml (with p=1.0) and the gamble (0.1 ml, p=0.5; 1.2 ml, p=0.5), the probability of getting of 1.2 ml was larger after choosing the safe option, compared to the gamble (p = 1 vs p = 0.5) and thus the safe option first order stochastically dominated the gamble. By contrast, for choices between low reward safe option of 0.1 ml and the gamble (0.1 ml, p=0.5; 1.2 ml, p=0.5), the gamble first order stochastically dominated the safe option. The low reward of 0.1 ml was common between them, but the probability of getting of 1.2 ml was larger after choosing the gamble (p=0.5 compared to p=0). (B) PEST procedure (Parameter Estimation through Sequential Testing). Red and blue traces show tests for gambles (0.1 ml, p=0.5; 0.4 ml, p=0.5) and (0.9 ml, p=0.5; 1.2 ml, p=0.5), respectively. The gambles remained unchanged throughout a PEST sequence, whereas the safe amount was adjusted based on the previous choice following the PEST protocol (Experimental Procedures). Each data point shows the safe value offered on that trial. The CE of each gamble was estimated by averaging across the final two safe rewards of each PEST sequence (n and n-1). (C) Incentive compatible assessment of certainty equivalents (CE). Top: Animals chose between a safe reward and either a low value gamble (0.1 ml, p=0.5; 0.4 ml, p=0.5) (red, inset at top) or a high value gamble (0.9 ml, p=0.5; 1.2 ml, p=0.5) (blue). The safe reward amount varied randomly on each trial between 0 and 1.2 ml, independently of the animals' previous choice. The curves were derived from logistic functions fitted to choice frequencies averaged over 30 trials per data point. For each gamble, the dotted vertical line indicates choice indifference (CE), and the solid vertical line indicates the gamble's EV. For the gamble with low EV (red), the CE was larger than the gamble's EV, indicating risk seeking behavior. In the gamble with high EV (blue) shows the opposite, indicating risk aversion. (D) Iterative fractile method for measuring utility under risk. Use of binary, equiprobable gambles for constructing utility functions from certainty equivalents (CE). In step 1, the CE of the gamble between 0.1 and 1.2 ml (each p=0.5) was measured using PEST (Experimental Procedures) (here CE=0.76 ml) which corresponds to utility of 0.5. In step 2, the CE of the gamble between 0.1 and 0.76 ml was measured (CE=0.57 ml), corresponding to utility of 0.25. In step 3, the CE of the gamble between 0.76 ml and 1.2 ml was measured (CE=0.85 ml), corresponding to utility of 0.75. (E) Construction of utility function by bisecting the utility scale until seven CE–utility pairs were obtained, using the fractile method shown in B. (F) Measurement of utility from choices of gambles with reward probabilities between 0.05 and 0.95 in monkey B. The animal chose between a pie-chart stimulus indicating 0.5 ml of reward with specific probability p (vertical stripes) and no-reward with 1-p (horizontal stripes) vs. a safe outcome indicated by horizontal bar (example shows p=0.25 reward and p=0.75 no-reward vs. 1.1 ml safe reward). (G) Convex utility function derived from choices shown in F. Although the observed curvature shown in

G might result from a combination of non-linear utility and probability distortion (to be investigated in a future study), this function demonstrates that the utility measurements are quasi-continuous in probability. Moreover, the shape of this function (measured between 0 and 0.5 ml) reflects the convex initial segment of the function measured between 0.1 and 1.2 ml, rather than a compressed version of the whole function.

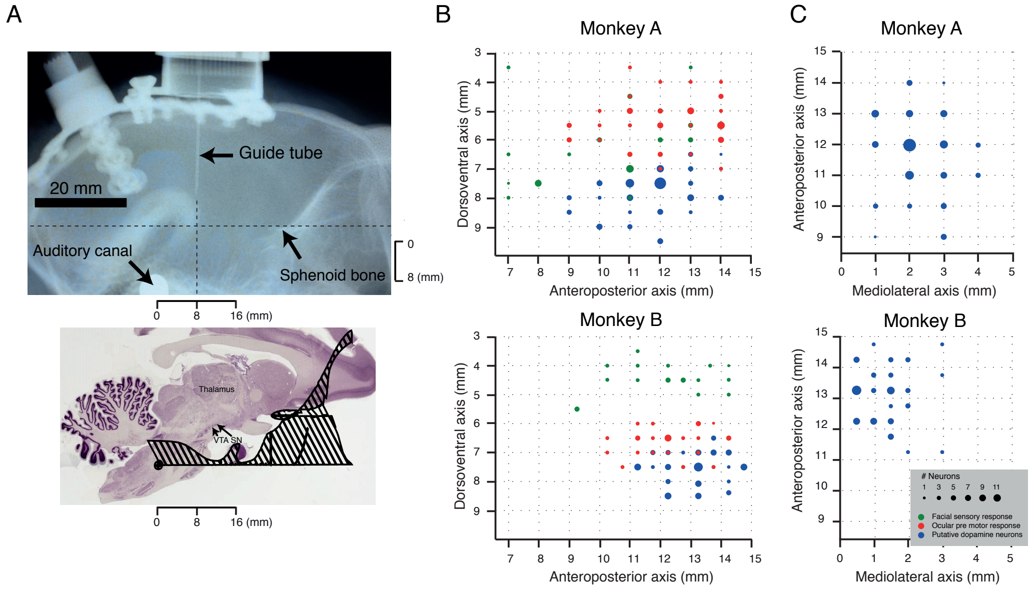

Figure S2: Recording sites of dopamine neurons (related to Figures 3 - 6)

(A) Top: X-ray of lateral view of monkey A's skull with a guide tube directed toward the midbrain area. Bottom: Composite figure of recording area in midbrain. The schematic drawing of the base of the skull was obtained from Aggleton and Passingham [S1]. Nissl-stained standard sagittal histological section from Macaca mulatta was obtained from www.brainmaps.org (slide 64/295). All figure components are displayed at the same scale as the X-ray shown in A, top. (B) Anteroposterior (relative to interaural line) and dorsoventral (relative to midline) view of the recording track in monkey A (Top) and monkey B (Bottom). Symbol sizes indicate numbers of neurons recorded in each track (right hemisphere in both animals). (C) Surface view of recording locations in monkey A (Top) and monkey B (Bottom) in mediolateral and anteroposterior axes.

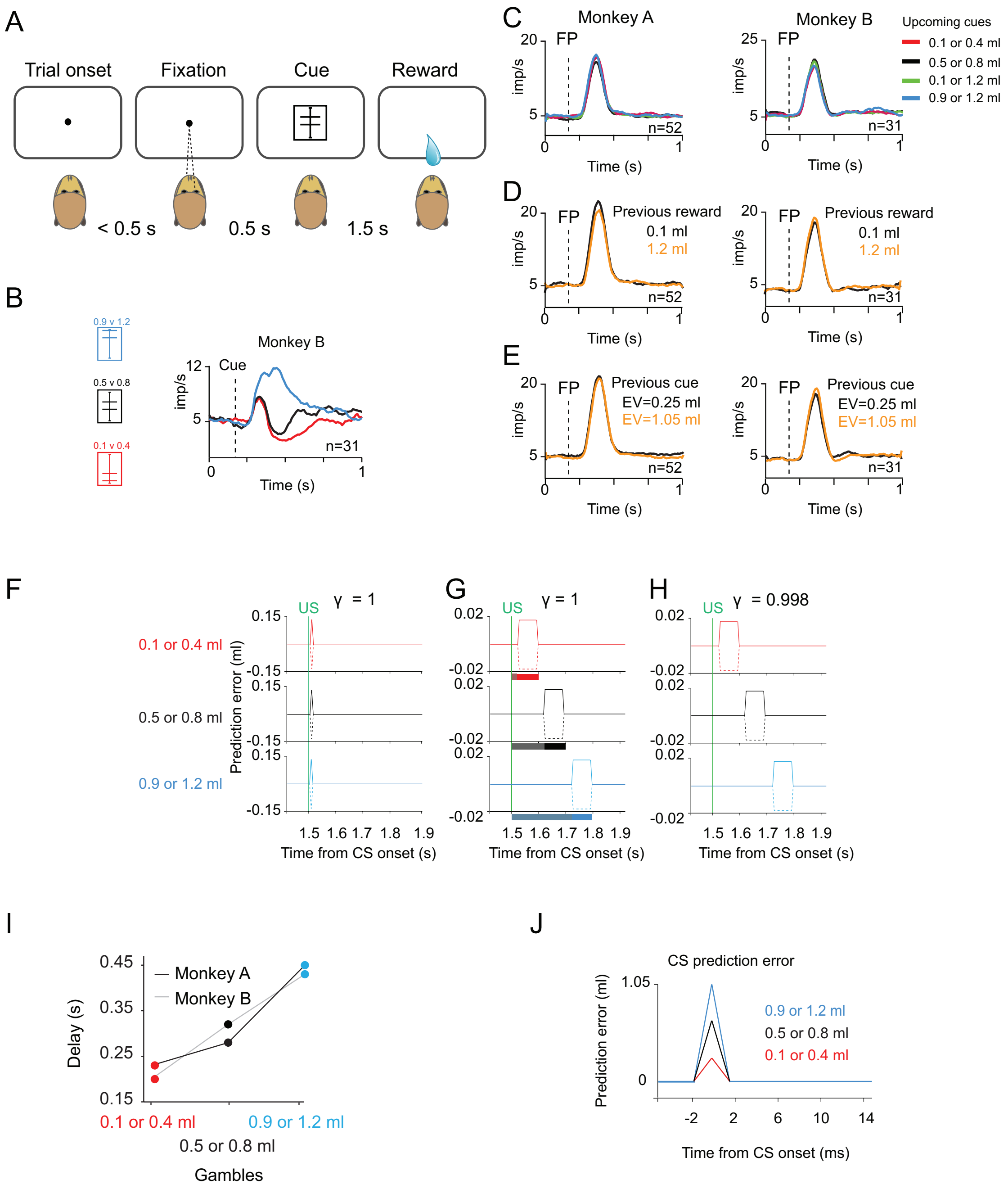

Figure S3: Non-choice task and additional dopamine responses (related to Figure 3)

(A) Sequence of trial events in the recording task. Each trial began with a fixation point at the center of the monitor. The animal directed its gaze to it and held it for 0.5 s. Then the fixation point disappeared and a cue predicting a gamble occurred. Gambles alternated pseudorandomly. The cue remained on the screen for 1.5 s. One of the two possible gamble outcomes was delivered at cue offset in pseudorandom order. Unsuccessful central fixation resulted in a 6 s time-out. There was no behavioral requirement after the central fixation time had ended. (B) Population responses in monkey B (n=31) to cues shown in B left ($p < 0.00001$, rho = 0.75; Pearson's correlation with gamble EV). (C-E) Constant dopamine responses to fixation point (FP). The FP predicted the constant mean reward value from all trial

types combined. (C) Population responses separated according to the upcoming gamble cues. These responses were not obviously modulated by reward or trial history (D) Population responses separated according to the reward delivered in the previous trial. (E) Population responses separated according to the cue presented in the previous trial. n = number of dopamine neurons. (F-H) Temporal difference (TD) modeling. Prediction errors to reward of fully trained TD models to 0.1 or 0.4 (top), 0.5 or 0.8 (middle), 0.9 or 1.2 ml (bottom), respectively. In F rewards were represented as volume delivered at reward time. In G and H rewards are shown as delivered during the open liquid solenoid valve. Horizontal bars in G indicate solenoid opening times for different reward sizes. γ is temporal discounting coefficient per 2 ms time bin. (I) Comparison of neuronal timing with TD model timing: Onset of differential dopamine reward responses for gambles with different EV. Onset is defined as the first temporal window (of 10 ms duration) after reward onset in which positive and negative prediction error responses are statistically different ($p < 0.05$, t-test). (J) Learned TD responses to cues predicting the three gambles of G-I following training with unpredicted rewards.

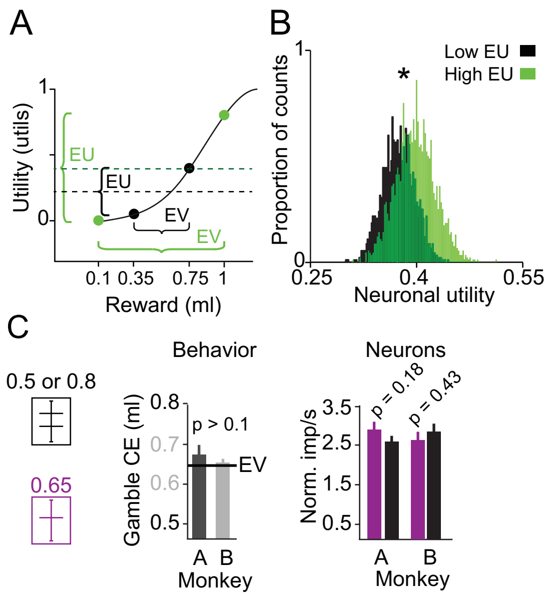

Figure S4: TD model in monkey B and additional neuronal responses (related to Figure 5)

(A) Gambles for reinforcement modeling in monkey B. Two gambles (0.1 ml, p=0.5; 1.0 ml, p=0.5) (green) and (0.35 ml, p=0.5; 0.75 ml, p=0.5) (black) had equal expected value (EV = 0.55 ml) but different risk. The gambles were aligned on the previously established utility function (Figure 1F) and yielded higher (green) and lower (black) expected utility (EU) (horizontal dashed lines). (B) Histograms of learned TD prediction errors reflect the expected utility of gambles when trained on neuronal responses from monkey B (Figure 4C). Each histogram is comprised of 2000 data points that represent the predicted gamble value (high expected utility gamble in green versus low expected utility gamble in black, defined in (A) following training with the neuronal responses from monkey B to the respective gamble outcomes (Figure 4C, * $p < 10^{-158}$; t-test). (C) Absence of direct risk coding in short latency dopamine response to reward-predicting cues. Left: Cues predicting safe reward (0.65 ml) or gamble (0.5 or 0.8 ml) placed on linear, risk neutral part of utility function. Center: Mean certainty equivalents (CE) for gamble assessed in choices. The proximity of gamble CE to gamble EV (horizontal line) confirms risk neutrality and suggests similar EU between gamble and safe reward. Right: Overlapping neuronal responses to cues predicting safe reward (purple, 0.65 ml) or gamble (black, 0.5 or 0.8 ml) of similar utility but different risk ($p > 0.18$ and 0.43, in monkeys A and B, respectively, t-test).

	X1 (ml)	X2 (ml)	EV	CE monkey A	CE monkey B
Gamble 1	0.1	0.4	0.25 ml	0.34 (±0.08) ml	0.32 (±0.06) ml
Gamble 2	0.1	0.5	0.3 ml	0.36 (±0.13) ml	0.43 (±0.09) ml
Gamble 3	0.2	0.6	0.4 ml	0.38 (±0.09) ml	0.49 (±0.08) ml
Gamble 4	0.3	0.6	0.45 ml	0.49 (±0.07) ml	0.54 (±0.07) ml
Gamble 5	0.3	0.7	0.5 ml	0.59 (±0.12) ml	0.56 (±0.12) ml
Gamble 6	0.4	0.8	0.6 ml	0.73 (±0.04) ml	0.66 (±0.11) ml
Gamble 7	0.5	0.8	0.65 ml	0.71 (±0.08) ml	0.71 (±0.12) ml
Gamble 8	0.5	0.9	0.7 ml	0.76 (±0.06) ml	0.79 (±0.11) ml
Gamble 9	0.6	1	0.8 ml	0.81 (±0.08) ml	0.82 (±0.09) ml
Gamble 10	0.7	1	0.85 ml	0.83 (±0.07) ml	0.88 (±0.04) ml
Gamble 11	0.7	1.1	0.9 ml	0.87 (±0.07) ml	0.88 (±0.05) ml
Gamble 12	0.9	1.2	1.05 ml	0.92 (±0.04) ml	0.91 (±0.04) ml

Table S1: Gambles for out of sample prediction (related to Figure 2)

Expected values (EV) and certainty equivalents (CE, mean ± 1 SD) of the 12 gambles used for out-of-sample prediction. X1 = outcome 1 of gamble, X2 = outcome 2 of gamble, both delivered with probability = 0.5.

Supplemental Experimental Procedures

Validating utility functions with different reward probabilities

Utility functions should be a continuous function of probability. To test whether our methodology to assess utility functions was robust to different probabilities, we also measured the CE of gambles that predicted reward with probabilities ranging from 0.05 to 0.95. Probability was conveyed using circular pie charts. They were divided into 2 striped regions whose areas indicated the probability of receiving 0.5 ml (vertical stripes) or no reward (horizontal stripes), respectively (Figure S1F). We measured the CE of these gambles using the PEST procedure and derived the utility. Similar to the utility functions in the small reward range measured with a fixed probability (p=0.5), the utility function measured with different probabilities was convex (Figure S1G).

Identification of dopamine neurons

Custom-made, movable, glass-insulated, platinum-plated tungsten microelectrodes were positioned inside a stainless steel guide cannula and advanced by an oil-driven micro-manipulator (Narishige). Action potentials from single neurons were amplified, filtered (band-pass 100 Hz to 3 kHz), and converted into digital pulses when passing an adjustable time-amplitude threshold (Bak Electronics Inc.). We stored both analog and digitized data on a computer using custom-made data collection software (Matlab). We recorded the extracellular activity of single dopamine neurons within the *substantia nigra* and in the ventral tegmental area (A8, A9 and A10). We localized the positions relative to the recording chamber using X-ray imaging and functional properties of surrounding cell groups (Figure S2). Post-mortem histology was postponed due to ongoing experiments with these animals. We rejected all neuronal recordings with < 10 trials per experimental condition.

Reinforcement model

Temporal difference (TD) models are standard reinforcement learning algorithms [S2]. We used a conventional TD model that consisted of a prediction error term, as follows:

$$\delta(t) = r(t) + \gamma * V(t+1) - V(t) \qquad \text{Eq. 1}$$

where t is time, r is reward, γ is the temporal discount factor, and V is predicted physical value or predicted utility. The prediction error term was used to update a value function:

$$V(t) \leftarrow V(t) + \alpha * \delta(t) \qquad \text{Eq. 2}$$

where α is the learning rate.

This model was used to assess temporal aspects of reward delivery (Figure 3, see below). It was also employed to demonstrate appropriate learning of expected utility, using an eligibility trace $\lambda = 0.9$ as before [S3] (Figure 5 and S4).

Modeling temporal aspects of reward delivery

We explored different variations of the TD model to ensure that the particular nature of the temporal prediction errors in the experiment did not explain the neuronal responses observed in Figure 3. We performed three different simulations. In the first

one, we represented the reward as the magnitude (in ml) at the time of reward onset. After training (we discarded the first 2000 trials), the model produced identical prediction errors to the rewards in the gambles between 0.1 and 0.4 ml (Figure S3F top), 0.5 and 0.8 ml (Figure S3F middle), and 0.9 and 1.2 ml (Figure S3F bottom). Thus, the model did not explain the response variations shown in Figure 3.

However, with our solenoid liquid valves, reward amounts were determined by valve opening times. Larger rewards required longer valve opening times. Therefore, following each reward-predicting gamble, the animal could only predict whether the larger or small reward was delivered after the valve opening duration for the smaller predicted reward. At this point, the liquid flow stopped with the smaller reward of the gamble (in half the trials), but continued for the gamble's larger reward (in the other half of the trials, indicated by the gray and colored bars below the traces in figure S3G). To account for these timing differences introduced by our reward delivery system, we performed two further simulations that emulated the solenoid reward delivery by representing the reward as a string of small units of 0.008 ml reward, each occurring in a time bin of 2 ms (our solenoids delivered 0.004 ml/ms). For example, a reward of 0.2 ml would be represented as a string of length 25, whereas a reward of 0.4 ml would be represented as a string of length 50. We ran the simulation with and without temporal discounting ($\gamma = 1$ in Figure S3G and $\gamma = 0.998$ in Figure S3H). The discount factor was meant to account for possible value differences that might have arisen because the prediction error occurred later for larger gambles (i.e. the prediction error for the gamble between 0.5 and 0.8 ml occurred later than the prediction error for the gamble between 0.1 and 0.4 ml). The small size of discount value was necessary because of the fine time bins used (2 ms). In these simulations, prediction errors occurred at the offset of the smaller reward in each gamble, and they occurred later for the larger gambles, mirroring the timing of the neuronal responses (Figure S3I). The model learned appropriately scaled cue responses for the three gambles (Figure S3J). However and importantly, the modeled prediction error responses failed to show the non-monotonic variation of the dopamine responses displayed in Figure 3C, D which reflected marginal utility. Thus, the nature of the prediction errors determined by the reward delivery system could not explain the nature of the neuronal responses.

Supplemental References

S1. Aggleton, J., and Passingham, R. (1981). Stereotaxic Surgery Under X-Ray Guidance in the Rhesus Monkey, with Special Reference to the Amygdala. Exp. Brain Res. *44*, 271–276.

S2. Sutton, R. S., and Barto, A. G. (1998). Reinforcement Learning: An Introduction (Cambridge, MA: The MIT Press).

S3. Pan, W.-X., Schmidt, R., Wickens, J. R., and Hyland, B. I. (2005). Dopamine cells respond to predicted events during classical conditioning: evidence for eligibility traces in the reward-learning network. J. Neurosci. *25*, 6235–42.
